# Integrate & balance aspects for safe and sustainable innovation: Needs analysis on SSbD categories and product development stage requirements to cover the entire life cycle

**DOI:** 10.1016/j.csbj.2025.07.030

**Published:** 2025-07-17

**Authors:** Gustavo Martin Larrea-Gallegos, Sabine Hofer, Norbert Hofstätter, Benjamin Punz, Nico Watzek, Wibke Lölsberg, Karin Wiench, Wendel Wohlleben, Irantzu Garmendia Aguirre, Nikolakopoulos Athanassios, Haralambos Sarimveis, Anna Costa, Christian Seitz, Steffi Friedrichs, Thomas E. Exner, Roland Hischier, Antonino Marvuglia, Martin Himly

**Affiliations:** a Environmental Sustainability and Circularity Assessment (SUSTAIN) Research Unit, Luxembourg Institute of Science and Technology, 5, avenue des Hauts-Fourneaux, Esch/Alzette L-4362, Luxembourg; b Dept. Biosciences & Medical Biology, Paris Lodron University of Salzburg (PLUS), Hellbrunnerstrasse 34, Salzburg 5020, Austria; c BASF SE, Carl-Bosch-Straße 38, Ludwigshafen am Rhein 67056, Germany; d European Commission, Joint Research Center (JRC), Ispra, Italy; e School of Chemical Engineering, National Technical University of Athens (NTUA), Zografou 15780, Greece; f Istituto di Scienza e Tecnologia dei Materiali Ceramici (CNR-ISSMC), Via Granarolo, 64, Faenza, RA 48018, Italy; g AcumenIST, Rue Fétis 19, Etterbeek 1040, Belgium; h Sevenpastnine GmbH, Rebacker 68, Schopfheim79650, Germany; i Technology & Society Laboratory, Swiss Federal Laboratories for Materials Science and Technology (EMPA), Lerchenfeldstrasse 5, St. Gallen 9014 , Switzerland

**Keywords:** Safe & sustainable by design, innovative advanced materials, life cycle assessment, machine learning, artificial intelligence, digitalization, FAIR data, multi-objective optimization, responsible research & innovation, SSbD framework, substitution chemicals, green chemistry

## Abstract

Current EU Strategies aim to rapidly advance the research, development and deployment of innovative advanced materials and chemicals to make Europe the first digitally enabled circular, climate-neutral and sustainable economy. To achieve this, an underlying adaptation of the research and innovation (R&I) process to the Safety-and-Sustainability-by-Design (SSbD) framework has been proposed. This perspective article provides an overview of already existing approaches providing guidance for implementing SSbD-like procedures in R&I in several different industrial sectors to ultimately replace substances of concern (SoC). Starting from the ECHA’s Assessment of Alternatives (AoA) approach we put emphasis on the scoping phase during which the requirements for replacement will be identified. The limitations for the changes possible and trade-offs acceptable for the company need to be defined, in agreement with relevant stakeholders to be further involved in AoA scoping (*e.g.* for setting the trade-off levels). This includes listing the SSbD-relevant aspects in the different categories (*i.e.* functional performance, health, environment, social, and economic sustainability) in a customized manner, followed by weighting them in relation to their expected impact on the intended SSbD-guided multi-objective optimization procedure. An additional dimension is provided as to how to deal with uncertainties (*e.g.* data gaps or compromises in data quality, or which assessment methods and tools to employ); notably, it represents the company’s own decision to herewith set the requirements and goals for replacement, and this can be done at different levels, such as the material or chemical itself, changes in the production processes, or within the entire system of a product’s life cycle spanning across its entire value chain(s), which can be documented employing the use maps concept. Further, this article builds on the product life cycle and provides a general understanding of life cycle assessment (LCA) methodology, especially a deeper insight into prospective and anticipatory LCA, that will need to prove functional on real-life case studies from industry. Besides a clarification of these concepts, the article provides an interdisciplinary view, as required for implementing SSbD in small and medium-sized enterprises, with hints on the use of machine learning techniques for anticipatory LCA of new chemicals, materials, and products. Such methodologies will, in future, help extend classical LCA cases towards the data-scarce requirements of earlier material and product development stages.

## Highlights

• Focus on industry requirements for implementing SSbD at early development stages.
• Employing computational workflows for SSbD integrating and balancing the dimensions functionality, safety, and sustainability.
• Overview on LCA and insight in current progress in prospective and anticipatory LCA procedures.
• Challenges and opportunities of machine-learning-assisted approaches to assess impact across the entire life cycle.
• SSbD framework in relation to previous risk assessment frameworks and industry practice.

## 1 Introduction

The European Green Deal (2021) binds the European Union (EU) to become climate neutral by 2050 through green growth and innovation, offering opportunities for all citizens and protecting biodiversity [1]. This gave rise to a number of European policies*,* such as the Chemicals Strategy for Sustainability (CSS) [2], Zero Pollution Action Plan [3], Circular Economy Action Plan [4], Biodiversity strategy [5], Critical Raw Materials Act [6], EU Goals for Air, Soil and Water [7], Farm to Fork strategy [8], or the Fit for 55 Package [9]. These policies call for a twin green and digital transition [10] towards a more sustainable, climate-neutral, circular, globally competitive and resilient economy. While such a kind of transition requires a multisectoral view, focusing on the chemicals and materials sectors is paramount due to their key role in most value chains. Improving these sectors requires research and innovation (R&I) so novel and enhanced functionalities can help to improve performance and replace the reliance on critical raw materials and substances of concern (SoC). However, this objective is not simple since newly developed innovative chemicals and advanced materials (ChAMs) need to be safe, sustainable and preferably circular while still maintaining the expected functionality. In this context, the *Safe-and-Sustainable-by-Design (SSbD)* concept [11], proposed by the European Commission (EC)’s Joint Research Centre (JRC), has been adopted as an EC recommendation (referred as EU SSbD Framework hereafter) in December 2022 [12].

Since its introduction, multiple efforts to operationalize the SSbD Framework have yielded a variety of EU-funded research projects that aim to accelerate the advancement of SSbD-oriented methodologies across multiple industry sectors and case studies [13]. Emerging challenges were identified as often being harboured within the intrinsic multidisciplinarity and -dimensionality of SSbD. In this sense, this perspective article presents an overview of the existing approaches providing guidance in the implementation of SSbD-like procedures in R&I. We dissect the main components of SSbD to expose our views, stressing the importance of focusing on early stages of the innovation process and adopting a full life cycle perspective. Our attention is on the challenge of **balancing** the different SSbD criteria, and how to **integrate** the right tools at adequate time. Moreover, we provide our perspective on the challenges and opportunities that the use of novel techniques, such as Machine Learning (ML) and Artificial Intelligence (AI), can bring as accelerators of SSbD operationalization, especially at early stages of innovation.

This article starts (2) by revisiting the main aspects of policies like CSS to then describe how the above-mentioned goals led to the introduction of the EU SSbD Framework as a voluntary approach for advanced materials innovation. Moreover, we discuss how the EC, the industry, the academia and regulatory stakeholders can work together to refine the SSbD Framework relying on the experiences gained from case studies to make it practicable and operational. 3, 4 then look at important concepts and new developments for safety and sustainability, respectively, to then give additional guidance on how to adopt a holistic view on the complete material life cycle of complex and circular value chains. 5 describes how `Safe` and `Sustainable` encounter more challenges when addressed in a `By-Design` context. This section finally describes how this is meant to be translated into the specific research and innovation actions of the EU-funded project PINK, which combines computational approaches to predict and optimize simultaneously functionality, economic feasibility, safety and sustainability throughout the complete materials development process from early ideation to market introduction.

## 2 Revisiting key information of the SSbD framework

### 2.1 The chemicals strategy for sustainability and the EU SSbD framework

The European economic, social and legislative environment has enabled and fostered a mentality in which innovation is seen as a balance among functionality, performance, cost, safety, and sustainability. By prioritizing sustainability in product portfolio management and continuous renewal, companies aim to protect the environment, address climate change, and preserve natural resources for future generations. In many companies, stepwise R&I processes are followed, such as stage-gate processes (*e.g.* Cooper *et al.* [14]), which have played a crucial role in achieving these objectives. However, the recent CSS policy has introduced more stringent requirements that include banning the most hazardous substances in consumer products unless their use is deemed essential, integrating the "cocktail effect" of chemical mixtures into risk assessments through the introduction of a mixture assessment factor, and phasing out per- and polyfluoroalkyl substances (PFAS) across the EU, with exemptions only for essential uses. The strategy also aims to stimulate investment and adoption of SSbD chemicals, enhance the security and sustainability of critical chemical supply chains, and streamline regulatory processes via a "one substance, one assessment" approach. Additionally, the EU commits to global leadership by promoting stringent safety standards and restricting the export of chemicals banned within the EU. In this context, the JRC created practical guidance on how to implement these requirements in chemicals and materials innovations in the form of the EU SSbD Framework, initially characterized by its holistic, precautionary, and highly formalized nature [11]. Following the typical definition of the industrial innovation processes along the Cooper’s stage-gate model [14], this Framework proposes two components: re-design phase of the chemical/material, process, or product/application (*i.e.* stage) and a 5-step assessment phase (*i.e.* gate).

In the original version of 2022, the re-design phase is mainly defined by a non-exhaustive list of design principles, which become relevant at different stages of the innovation process of a given product and guide R&I towards safer and more sustainable alternatives. It takes inspiration from green chemistry, green engineering, circular and sustainable chemistry, and systems that minimize their environmental impact and promote sustainability [15], [16], [17]. Some key design principles associated with these fields are:

#### 2.1.1 Green chemistry

• Atom Economy: Aim to maximize the incorporation of all materials used in a process into the final product, minimizing waste and by-products.
• Non-toxic Products: Avoid or minimize the use of toxic substances in the design and synthesis of chemicals, ensuring that the end products are safer for human health and the environment.
• Design for Degradation: Design products and materials that are easily degradable, reducing the persistence of pollutants in the environment.
• Green Catalysts: Use environmentally friendly catalysts that are efficient and produce minimal waste in chemical reactions.
• Sustainable Synthesis Routes: Choose synthetic routes that are environmentally benign, employing methodologies that reduce the use of hazardous substances and energy.

#### 2.1.2 Green engineering

• Energy Efficiency: Design processes and systems to be energy-efficient, utilizing renewable energy sources whenever possible to reduce overall environmental impact.
• Reduced Emissions: Minimize emissions of pollutants and greenhouse gases during the manufacturing and use of products or in industrial processes.
• Water Conservation: Implement water-efficient processes and technologies to minimize water consumption and reduce water pollution.
• Process Intensification: Optimize processes to be more compact, efficient, and resource-effective, reducing the overall environmental footprint.

#### 2.1.3 Circular and sustainable chemistry

• Renewable Resources: Prioritize the use of renewable feedstocks and resources to reduce dependence on finite resources and minimize environmental impact.
• Life Cycle Assessment (LCA): Consider the entire life cycle of a product, from raw material extraction to manufacturing, use, and disposal, to assess and minimize environmental impacts at every stage.
• Biodegradable Materials: Design materials that are biodegradable, reducing the environmental impact of waste disposal.
• Circular Economy: Embrace circular economy principles, promoting the reuse, recycling, and recovery of materials to minimize waste and maximize resource efficiency.
• Social Responsibility: Consider the social aspects of sustainability, including the well-being of workers, local communities, and the global population.

The gate phase is composed of steps for (1) hazard assessment, (2) human health and safety aspects in the production and processing phase, (3) human health and environmental aspects in the final application phase, and (4) environmental sustainability assessment, which can be complemented with (5) an optional socio-economic step [18]. Steps 1–4 are the further defined by a large set of assessment factors and indicators, which need to be considered when making the decision to continue with a specific material design idea to the next development stage. It is important to note here that a considerable number of hazard endpoints such as those that could negatively impact the sale of the new product (*e.g.,* Substance of Very high Concern (SVHC) criteria: carcinogenic-mutagenic-reprotoxic (CMR); persistent, bioaccumulative, toxic (PBT); very persistent, very bioaccumulative (vPvB); persistent, mobile, toxic (PMT); very persistent, very mobile (vPvM) environmental and human endocrine disruption (ED)) are labelled as hard stop criteria. This means that the insufficient performance for these indicators can trigger a direct removal of the ChAMs from the list of design candidates. In contrast, it is difficult to identify hard stop criteria for sustainability-related indicators (*i.e.* LCA impacts) since they are originally designed for relative comparison, meaning that specific decision heuristics would need to be designed.

### 2.2 The role of the chemical industry in SSbD

Even when there is a general agreement by industry that SSbD is needed to address global challenges, criticism was still pointed at issues regarding the practicability and manageability of the framework [19] as well as the selection of hazard instead of exposure as the primary component of safety assessment. To transform this criticism into constructive propositions, the European Chemical Industry Council (CEFIC) and its members provided a re-interpretation of the SSbD concept and defined guidelines for implementing SSbD as an approach to (industrial) innovation processes [20], which will be shortly recapitulated here.

During the development, great importance was attached to aligning this industry view with the broader goals of the European Green Deal and the CSS. However, it also expressed that there should be a clear strategic link between the application of any SSbD framework and the purpose of research and innovation steering, including potential incentives for implementing SSbD frameworks. The complementary use of SSbD frameworks with established approaches for portfolio analysis, such as the Product Sustainability Assessment (PSA) of the World Business Council for Sustainable Development (WBCSD), is seen as synergistic and advantageous. CEFIC built its understanding of SSbD principles taking into account views/perspectives of other organizations, such as the OECD, the UN Sustainable Development Goals and the WBCSD, as SSbD approaches implemented in industrial innovation processes should prioritize lean and pragmatic strategies [21]. CEFIC also believes that criteria for chemicals that are SSbD must address the three pillars of sustainability – environmental, social and economic factors – and take a life cycle approach. Actions following those principles will drive the shift towards a circular economy and climate-neutral society.

**Fig. 1:**
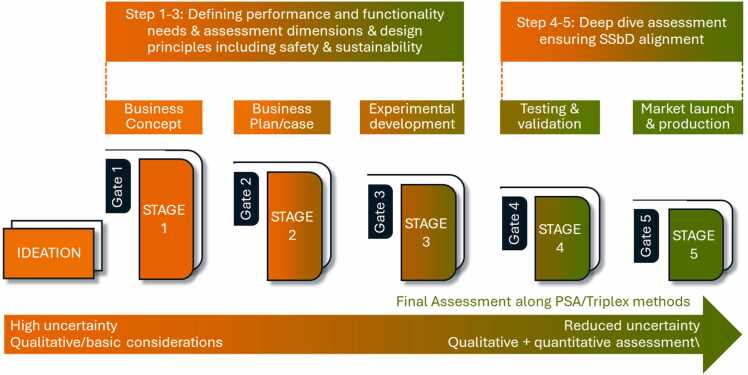
Schematic illustration of an example of an innovation process (*e.g.* stage-gate process, illustrating the increasing level of certainty during the assessment of safety and sustainability).

Adapted from [20].

Industry’s view is aligned with the EU SSbD Framework when considering that SSbD should preferably be integrated into the R&I phase of all new materials and products by employing a set of guiding principles and assessing the product-application combination through an exemplary stage-gate approach. In brief, the stage-gate (Fig. 1) approach typically begins with ideation (up to Gate 3), in which business opportunities including feasibility analyses are evaluated [14]. This involves generating ideas to create new possibilities, solve problems, and identify potential opportunities. The next step is the conceptualization, where alternative ways of understanding and defining a problem or opportunity are explored, along with offering ideas to address it. Following this, business planning takes place, and experimental work in the laboratory is conducted to test and evaluate different concept candidates against targeted innovation goals. The novel substances are produced at lab-scale (up to Gate 4) and later up-scaling is performed (up to Gate 5), both representing the innovation development. Through ongoing validation, the number of suitable candidates is narrowed down. If one of the candidates successfully meets all innovation goals, including safety, sustainability, and economic viability, it proceeds to the launch phase and is introduced to the market. However, it is common for the innovation process to involve iterations and feedback loops, with continuous refinement and revisiting of earlier steps before reaching the final stage.

When comparing this process with the list of design principles of the EU SSbD Framework listed above, one can see that some of the principles naturally come into play at later stages when details on the industrial process are available (*e.g.* design principles Energy Efficiency, Reduced Emissions, LCA, Process Intensification), while design principles Renewable Resources, Non-toxic Products, or Design for Degradation, for instance, can be addressed right from the start, when choosing the type of raw material.

We argue that to not harm competitiveness, the necessary resources and capacities for SSbD application should not extensively increase in resources nowadays allocated to innovation projects. To make this possible, further development of flexible and adaptable assessment methodologies, such as digital predictive approaches like modelling, as well as analytical methods and toolboxes with comprehensive databases, is required now to facilitate the development of new molecules, materials, products, processes, and services for substitution or new applications. In other words, developing the needed assessment methods and providing them to the industry is essential to achieve practicability and manageability of the SSbD Framework. As it can be expected, this requires appropriate funding instruments and methods, including funding for academic partners and industrial bodies. Additionally, simplified, and accelerated approval procedures for newly developed SSbD products should be considered. More broadly, "market pull" measures to encourage safe and sustainable products and facilitate the transition of chemical companies to low-carbon and circular products could also be implemented [22].

Availability and accessibility of data throughout the value chain of chemicals and materials play another crucial role. Secure approaches to data sharing and trusted and federated data sharing spaces need to be developed, respecting the protection of intellectual property. To maintain the global competitiveness of the EU chemical industry, it is crucial to avoid creating isolated European solutions for SSbD. Implementing market pull measures without a cost framework that allows EU industry to stay competitive may attract non-EU producers who can provide similar functionality at lower costs. This issue is exacerbated by industry support mechanisms in other regions, such as tax credits and subsidies. Therefore, market pull measures should be integrated into a comprehensive framework to enhance Europe's global competitiveness. Incentives on the market side are effective only when production-side support has been established, including competitive energy prices, regulatory simplification, funding, and access to feedstock and raw materials. Is then under these conditions that the demand side can contribute to the return on investment.

### 2.3 Working together for a manageable SSbD

To further address the challenges posed by this framework, companies have established task forces comprising experts from various areas to assess the practicality of implementing the SSbD framework for both established products and new chemicals, materials, and processes. Simultaneously, the EU started opening specific SSbD calls for funding and launched two testing periods of the Framework, where it encouraged companies and public-private consortia to provide feedback on the application of the SSbD principles to real-world case studies. This resulted in the implementation of SSbD in R&I projects within industry often part of the publicly funded projects like ASINA, BIO-SUSHY, and then the projects funded under the HORIZON-CL4–2023-RESILIENCE-01 calls for proposals including the PINK project. In May 2024, the JRC published a new guidance based on the results from the first testing period (*i.e.* May-June 2023), acknowledging the difficulties in implementing the originally proposed SSbD Framework and recognizing the need for simplified approaches to SSbD in early innovation stages [18]. This focus on practicability and manageability is expected to be even more pronounced in the completely new version of the SSbD Framework, currently drafted and based on the results of the second testing period (*i.e.* May-August 2024).

The 2024 updates recognize more explicitly that substance or material development does not follow a linear trajectory. Rather, it proceeds through an iterative cycle. This cycle involves generating new evidence by testing for critical properties, such as (eco)toxicological characteristics, and making informed decisions on how to advance. The new evidence collected in subsequent cycles should further refine the selection process, ultimately leading to the development of a substance or material that optimally balances functionality or performance and economic viability with safety and sustainability. The SSbD material and product development process can thus be understood as defining and achieving optimal material performance, with performance defined by all the three pillars of sustainability – environmental, social and economic. Technically, this can be expressed as a multi-objective optimization problem that balances the above-mentioned design dimensions - functionality or technical performance, cost, safety, along with environmental and social sustainability - even if the exact balance might not be defined yet and might be case-dependent.

Moreover, it must be considered in a practicable SSbD approach that industry's search for new substances aims to minimize the likelihood of failure in later development stages, aligning with the ”fail early, fail cheap” principle. In the context of hazard and exposure assessment, it is crucial to recognize that conducting a comprehensive risk assessment — which entails evaluating all potential hazards and exposures to formulate protective measures and risk management strategies, including warnings, personal protective measures, or restrictions and bans to mitigate risk to an acceptable level — is not practical for all development candidates. Instead, the most effective approach is to assess the most critical properties of a substance at an early stage of development.

**Fig. 2:**
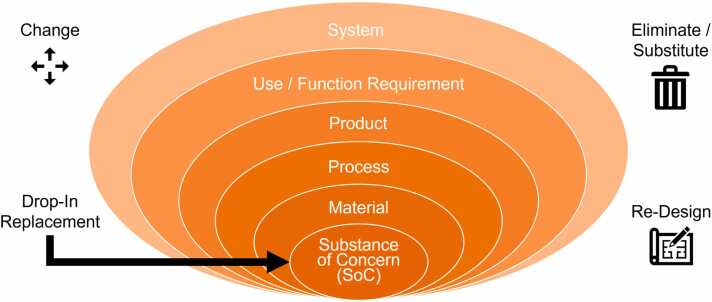
Shell diagram depicting the different possible layers where SSbD guidance can leverage safer and more sustainable R&I in industry and beyond extending from changing, eliminating, substituting, or redesigning elements from the substance of concern (SoC) to the system layer.

Additionally, the question of where to implement changes leading to safer and more sustainable products is inherent to the concept of SSbD and to an intended SSbD-guided optimization initiative in product development. “Where” refers here to levels of complexity, at which R&I investments can or need to be targeted and which can be described as an onion with the layers depicted in Fig. 2. Effectively, the innovation can be applied to each of the layers with different companies and value chain stakeholders involved. In its easiest form, the development only considers modifications at the SoC or material level, representing the concept of drop-in replacement. However, such one-to-one replacements are often not practical since even only changing one starting material will have an influence on the performance of the advanced material and/or the product, how they are produced and how they need to be treated at the end of life. Therefore, the perspective has often to be extended to include the product and underlying production processes, holistically considering the entire life cycle. The product level is also often the selected level since it might open possibilities to achieve a higher degree of safety, sustainability, and innovation. Going beyond, questioning the use or functional requirement or even the need for a specific technology (system level) may optimize the socio-economic impact of SSbD. At these highest levels (use/function requirement or system layer), innovators can reconsider the use (by asking the question of essential use) of the respective product or service and thus foster the development of completely new products or establishment of new industries. For instance, a system change towards more sustainable means of transportation may be implemented in the mobility sector or essential use may be reflected by eliminating (PFAS-based) coatings of cellulose-based one-way coffee cups in the packaging sector.

### 2.4 What next?

It can be concluded from the above, that SSbD will provide the most benefit when it is applied to include as many layers as possible providing a holistic view on the material life cycle and value chain on which design decisions can be based. It is also clear that all the information for such a holistic view will not be available at the early stages of the development and a manageable SSbD Framework needs to be based on a process, where early decisions can already be made based on a limited amount of information (fail early) and additional information can be integrated during the execution of the iterative development cycle. However, there is still uncertainty on how to best implement such a workflow customisable to the specific needs of the material development project as well as to the current material development stage (Technology Readiness Level (TRL) of the material), *i.e.* how to balance the different SSbD criteria according to the development goals and design stage, and how to provide the necessary tool set to support decision making at all these stages, *i.e.* integrate the right tools at the right time.

The ideas to address these challenges presented in this perspective article are based on the innovation plans of the PINK Project (*“Provision of Integrated Computational Approaches for Addressing New Market Goals for the Introduction of Safe-and-Sustainable-by-Design Chemicals and Materials”*) and were initially compiled as part of a training session on integrating and balancing assets for safe and sustainable innovation, in respect to different product development stages and in agreement with different SSbD dimensions including functionality [23]. PINK takes an industry-focused approach towards application of SSbD at the earliest possible stage by combining computational models and a decision support system (DSS) that harness the combined power of first-principles simulation and pre-existing data and knowledge. However, the perspective is going beyond the PINK project and is meant to provide fruit for thought for fostering a transition from a cradle-to-gate considerations currently still often adopted by material-developing industry to a cradle-to-grave or even a cradle-to-cradle view, in line with SSbD Framework’s holistic perspective on the entire material/product life cycle potentially even extending to circularity. In the next two sections, we will elaborate on what such a transition implies separately for the safety and the sustainability dimensions, and which remaining challenges must be overcome before we discuss solutions to be provided by the PINK project to bring everything together in a “by-design” approach.

## 3 S as in safety

Safety assessment as proposed to be executed within the first three steps of the EU SSbD Framework follows rather well-defined rules. Therefore, we will not review these here but first outline the general principles of comprehensive risk assessment based on mechanistic understanding, with focus on the concepts of New Approach Methodologies (NAMs), Integrated Approaches to Testing and Assessment (IATA), and Adverse Outcome Pathways (AOPs) (2.1) and then focus on preceding and meanwhile readily established methodologies, highlighting the Assessment of Alternatives (AoA) approach listed by ECHA, which goes beyond risk assessment of pristine materials, as currently demanded by regulations, by balancing risk with other business-related criteria like economic feasibility and availability of competences (2.2) and/or integrating specific use scenarios to cover more of the material life cycle (2.3).

### 3.1 Tiered risk assessment paralleling the material development cycle

**Fig. 3:**
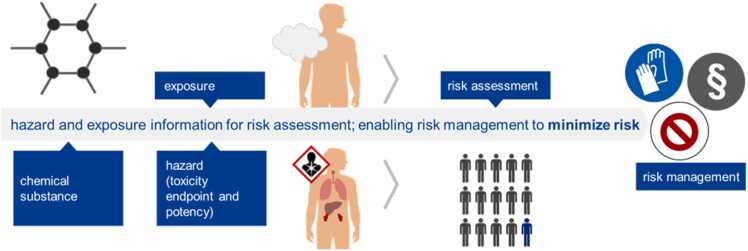
Schematic illustration of risk assessment. Information on a specific hazard (including the type and potency of toxic effects) as well as external and internal exposure is vital for assessing the likelihood and severity of potential harm to human health and for establishing suitable risk mitigation procedures.

Adapted from [24].

The general principle of risk assessment is depicted in Fig. 3 [24]. However, business goals, regulations as well as, and perhaps even more importantly, the availability and producibility of data make demands and set boundaries on which hazard endpoints and exposure scenarios can be included in an as-comprehensive-as-possible risk assessment at a given material development stage.

The most critical properties of a substance are defined as those that represent market cut-offs. While it is evident that a newly developed substance or material should not meet the criteria for SVHC, achieving this goal is more complex than it may seem. This is partly caused by the fact that not all SVHC criteria are straightforward to assess for all chemical classes. Additionally, depending on the market in which a new substance or material is intended to be introduced, other hazard classes - such as acute toxicity, skin sensitization, or even skin/eye irritation - may also impose market cut-offs. A general rule of thumb is that the more sensitive the intended application, the more critical negative Globally Harmonized System (GHS) classifications become. Considering such applicability and relevant issues of the test methods, it is practically impossible to establish a general or holistic testing strategy applicable to all development candidates. Furthermore, the sheer number of possible candidates that must be assessed during the early stages renders it economically and temporally unfeasible to test all substances simultaneously. Consequently, there is a growing understanding of the necessity to develop simplified, tiered, and targeted approaches specifically tailored for early R&I phases. As a result, companies are placing increased emphasis on projects and collaborating with internal and external partners to contribute methods and concepts that enable more efficient implementation of SSbD. This includes early hazard screening through analytics of synthesized structures and impurities using *in silico*, *in chemico*, or *in vitro* approaches, summarized as NAMs that can be utilized individually or in combination with other methods. They improve chemical safety assessments by providing more protective and relevant models, thereby contributing to the replacement of animal testing.

Significant progress has been made in adopting NAMs to assess chemical safety over the past 20 years. NAMs have proven effective for local toxicity endpoints like skin corrosion/irritation, eye damage/irritation, and skin sensitization, or also for mutagenicity/genotoxicity. However, these endpoints are driven solely by chemical reactivity or physicochemical properties. Defined Approaches (DAs) combining data of different sources such as *in silico*, *in chemico*, or *in vitro* with fixed interpretation procedures have facilitated regulatory use of *in vitro* methods.

Their value is acknowledged by organizations such as the OECD and has been integrated into IATA frameworks. In line with this, NAMs were combined with *in chemico* and *in vitro* studies to ultimately build AOPs. As an example, skin sensitization has been comprehensively addressed by the OECD, which established three mechanistically-based *in chemico* and *in vitro* test guidelines (TG) to assess the hazard potential of chemicals concerning the first three key events of the corresponding AOP: OECD 442 C [25], OECD 442D [26] and OECD 442E [27]. These methods were later incorporated into OECD 497 “DAs for Skin Sensitization” [28], showing that combining several NAMs can overcome the limitations of any single *in vitro* method. As animal tests historically targeted specific chemically induced adverse health effects in humans, available human data helped to validate NAMs approaches, which now follow OECD test guidelines widely referenced in many regulations globally.

Although efforts to develop NAMs have been substantial over the past decade, their implementation in regulatory toxicology has progressed slowly. Most often, the data generated by NAMs alone are not currently regarded as adequate by the regulatory community to determine a wide range of chemical safety-related endpoints for various substances, including industrial chemicals, plant protection products, cosmetics, and pharmaceuticals. The slow adoption of NAMs into regulatory processes is largely due to legislative frameworks that prioritize animal-based testing paradigms. For example, the standard information requirements (SIRs) under REACH for complex apical endpoints such as repeated dose toxicity or reproductive toxicity mandate specific animal test designs for hazard identification and characterization. Therefore, despite these advancements, animal testing remains heavily relied upon and is often mandated by law (*e.g.* Regulation (EC) 1907/2006 [29]). Using a set of NAMs to comply with the SIRs for a sub-chronic 90-day toxicity study requires generating comparable information typically obtained from the standard animal study [30]. It is necessary to cover all organs or systems and key parameters of an OECD 408 study, along with a sub-chronic 90-day exposure duration, and to derive a reliable quantitative Point of Departure (PoD). This may be addressed by using multiple NAMs together, each monitoring a key event in the underlying AOPs. However, registrants submitting such an approach must demonstrate how its results are sufficient for classification and labelling. For complex endpoints, this is challenging because current criteria for classification and labelling under the Globally Harmonized System of the United Nations (UN GHS) and its European implementing Regulation (EC) 1272/2008 [31] rely on data obtained either in humans, *in vivo* or from animal tests according to established OECD test guidelines or other internationally accepted test methods. Such NAMs do not provide the same hazard information as in comparison to traditional animal studies currently utilized and often required by regulations, it is important to acknowledge that investing in NAMs can involve significant financial costs and uncertainties. This investment may potentially lead to a loss if a safety decision cannot be made solely based on NAM data or if it is not accepted by regulators. Uncertainty regarding regulatory acceptance affects industries, particularly small and medium enterprises (SMEs), which face difficulties investing substantial resources in developing new products using NAMs if these approaches are rejected by regulators in certain sectors and jurisdictions due to legal requirements. Due to this reason animal testing provides clarity and legal certainty for the stakeholders involved [32], [33].

Nevertheless, employed in regulations (*e.g.* REACH), although some can be designed to yield similar classifications. However, historically used methodologies may become inadequate for future needs. NAMs can provide valuable mechanistic information that should be utilized directly for risk assessments or to prioritize substances for individual evaluations. Although these methods do not allow for absolute risk assessment, they enable comparative assessments between different versions of new chemicals or materials generated during the lab phase, benchmarked against conventional solutions. Moreover, NAMs not only serve as alternative non-animal approaches but can also be combined with *in vivo* test methods: for instance, gathering omics data within a short-term rodent bioassay, provides insights into pathways of disturbance and facilitates decision-making based on experiments with fewer animals or shorter duration, or can even be used to support scientifically based grouping or read across. Omics technologies generate large-scale molecular data, enhancing safety assessments. By studying mRNA (transcriptomics), proteins (proteomics), and metabolites (metabolomics), early signs of toxicity can be identified [34]. Metabolomics, particularly in biofluids like urine and blood, helps detect systemic changes across the organism after treatment [35]. Metabolomics has been employed to identify biomarkers associated with disease states, drug effects, and toxicity [36]. Additionally, metabolite profiling can facilitate pattern recognition approaches, where the collective response of multiple signals is utilized to characterize a specific state or reaction [37]. These pattern recognition methods achieve the highest accuracy when reference patterns are derived from an extensive database of profiles collected under controlled conditions [38]. For instance, a case study involving metabolomics data from 28-day studies in rats exposed to phenoxy herbicides demonstrated that a biological-based approach could be effectively used to select the optimal read across option among various candidates, thereby eliminating the need for a 90-day study as discussed above [39].

### 3.2 Assessment of alternatives for decision support

**Fig. 4:**
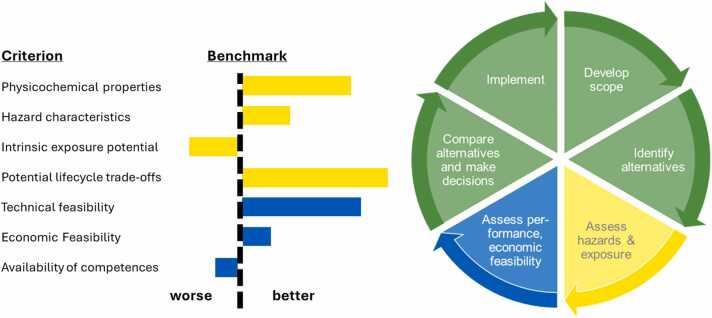
Schematic illustration of a guided circular approach to an assessment of alternatives (AoA), in which six steps foster a structured approach to implement an informed substitution: the assessment of hazard and exposure (yellow) and technical and economic performance (blue) inform comparison and decision making. Grading is done for each case-specific criterion relative to the SoC as the benchmark (red dotted line; worse, better).

The European Chemicals Agency lists AoA as one approach for listed as one approach for industry when registering products or for out-phasing SoCs. Originally, the term AoA, as currently used also by ECHA in their online training program, was coined by the Massachusetts Toxics Use Reduction Institute (TURI), summarizing “a process for identifying, comparing and selecting safer alternatives to chemicals of concern (including those in materials, processes or technologies) on the basis of their hazards, performance, and economic viability.” Nowadays, a nuanced approach of AoA includes modifications to processes or product redesign that facilitate the shift to safer processes and products [40]. Even beyond the product level, regulators, NGOs, and nonprofit organizations encourage industry to explore options for a change on system level, by questioning the need of function, rendering the SoC and its potential chemical alternatives non-essential (Fig. 2). With increasing awareness of the need to replace SoCs, driven by corporate governance, regulatory policies as well as arising societal and political concerns, an increasing number of AoA frameworks have been published and supporting tools implemented in recent years. Frameworks may cover very specific use cases, lacking a broader scope and variability. These original frameworks were designed to explore drop-in replacements using less hazardous substances. In contrast, holistic approaches define that AoA-frameworks have to cover six core elements, namely hazard assessment, technical feasibility, economic feasibility, exposure characterization, life cycle impacts and decision making [41]. In practice, the multidisciplinary nature of AoA requires a guided and orchestrated approach for stakeholders, which fosters scoping (problem framing), alternative identification, assessment of hazard and exposure, assessment of technical and economic feasibility, final decision making (comparison rules including trade off considerations) and ending up with successful implementation (Fig. 4).

#### 3.2.1 Scoping phase

Decisions must be made in every step of AoA, however, deciding on the boundaries in the scoping phase is critical and paves the way for potential final outcomes. From a specific company’s perspective, a reflection on the goals, corporate guidelines and principles, including decision rules and criteria is essential. Methodical approaches to all further assessment steps need to be determined and documented. Broad stakeholder participation in a collaborative manner fosters a right-sized and less biased framing [20]. However, stakeholder engagement may be challenging and time-consuming. A thorough characterization of the SoC, including physical-chemical properties, the function it serves in the specific application or product, the acceptable performance requirements and possible trade-offs is indispensable. Intrinsic exposure pathways as well as intrinsic hazard potential must be elucidated at this time. Notably, data on life cycle impacts would be desirable; however, they are frequently unavailable.

#### 3.2.2 Identify alternatives

Adopting a broader perspective is essential to fully exploit potential candidate options in scope, going beyond mere substitution of the SoC, i.e. extending to elimination, re-design and change on all levels of the SoC’s downstream engagement (outer layers in Fig. 2). Ample resources are available to support the search for suitable alternatives avoiding chemicals of concern or candidates with legal and authoritative issues (Table 1). Feasibility considerations in all its aspects, in particular technical or economic feasibility as well as market availability criteria, should not be used to prematurely screen out conceivable options. To strike the balance between the number of eligible alternative options and the feasible effort to manoeuvre the condensation to short-listed candidates, known bad actors and out-of-scope candidates, *e.g.* due to potential conflict to corporate goals, principles and guidelines as well as chemicals of emerging concerns, should be screened out in a documented and transparent manner. Obvious drop-in replacements to serve the same function without reconsidering the broader context should be regarded with suspicion, as experience has shown that drop-in replacements are frequently members of the same class of chemicals, such as PFAS, also referred to as “forever chemicals” [42]. As such, these may also share the adverse properties of the SoC, ending up in a regrettable substitution, a change from a known hazardous to not-yet-fully known and under-researched in class substance with incomplete data, as learned by the replacement of Bisphenol A by Bisphenol S [43].

**Table 1:** List of resources supporting the search for suitable alternatives, checking for SoC and avoiding candidates with legal and authoritative issues. NOTE: Some entries in this table are identical to the entries in other tables below, due to the nature of the respective listings.

**Resource**	**Provider**	**Description**	**Link**
ChemSec Marketplace	International Chemical Secretariat – ChemSec	Repository for green chemistry innovations (categories: PFAS, construction, electronics, textile; platform for buyers and sellers of alternatives to hazardous chemicals	https://marketplace.chemsec.org/
C&L Inventory	European Chemicals Agency (ECHA)	Classification and labelling inventory database containing classification and labelling information on notified and registered substances received from manufacturers and importers	https://echa.europa.eu/information-on-chemicals/cl-inventory-database
CleanerSolutions Database	Toxics Use Reduction Institute's (TURI) cleaning lab	Database which links performance evaluations to specific testing parameters and environmental assessments performed at the lab (categories: contaminants, substrates, equipment, solvents)	https://www.cleanersolutions.org/
ECHA CHEM	European Chemicals Agency (ECHA)	ECHA’s public chemicals database with information from all REACH registrations received by the Agency	https://chem.echa.europa.eu
3E former MyChemicalMonitoring	3E	Newly enhanced regulatory news; Understand your obligations, upcoming deadlines, and best practices	https://www.3eco.com/
Pharos	Habitable	provides hazard, use, and exposure information on more than 200,000 chemicals used in the materials economy	https://pharos.habitablefuture.org/
OECD Substitution and Alternatives Assessment Toolbox (SAAT)	OECD	Compiles resources (tools, data sources, case studies assessed by manufacturers, academic institutions, NGOs and government bodies) relevant to chemical substitution, selection and alternatives assessment.	https://www.oecd.org/en/topics/risk-management-risk-reduction-and-sustainable-chemistry.html
Substitution Support Portal (SUBSPORTplus)	Federal Institute for Occupational Safety and Health (BAuA)	Provides information supporting substitution of hazardous substances for a limited number of chemicals of concern in specific applications	https://www.subsportplus.eu/subsportplus/
U.S. Environmental Protection Agency’s Safer Chemical Ingredients List (SCIL)	United States Environmental Protection Agency (EPA)	List of chemical ingredients, arranged by functional-use class, that the Safer Choice Program has evaluated and determined to be safer than traditional chemical ingredients	https://www.epa.gov/saferchoice/safer-ingredients
WIPO GREEN	World Intellectual Property Organisation	Online platform / marketplace for technology exchange. It consists online database and network for bringing together a wide range of players	https://www3.wipo.int/wipogreen/

#### 3.2.3 Hazard, exposure and life cycle assessment

Chemicals management of safer alternatives to SoCs for a specific functional use requires the application of risk assessment models. Tiered approaches allow to circumvent data gaps and low confidence levels by making the resulting limitations and uncertainties and their influence on weighting and decision making transparent. Hazard and exposure assessment in AoA are comparative only, balancing known qualitative differences in human health toxicity and ecotoxicity relative to the benchmark material, thus allowing it to be based on incomplete data. Moreover, it focuses on the materials’ intrinsic hazard properties and associated exposure routes only, which are derived from known physicochemical properties of the material. Hazard assessment involves the characterization of a chemical alternatives’ inherent hazard properties with dimensions covering human health toxicity, ecotoxicity and physicochemical hazard endpoints. A gradual approach, starting with resources such as safety data sheets and the use of the GHS for hazard classification and labelling up to the adoption of predictive tools such as Quantitative Structure Activity Relationship (QSAR) may help to reduce costs and animal testing. The Organization for Economic Co-operation and Development (OECD), in close cooperation with the European Chemical Agency (ECHA), endorses the use of a QSAR software application [44]. Table 2 summarizes other existing resources to support hazard and exposure assessment. Most existing AoA frameworks implement a hazard-based assessment process only and underemphasize the need for integration of exposure dimensions in the context of the chemicals’ life cycle [41], [45]. Due to the scarce availability of exposure data, the weighting of exposure levels and potential exposure routes is commonly limited to a qualitative scheme. Comprehensive life cycle thinking along the alternative material’s potential value chain needs to be established and its potential consequences assessed, starting with material extraction, manufacturing, use and up to the end-of-life fate. Environmental aspects should be considered, in particular persistence, lack of (bio)degradability, bioaccumulation and the chemicals’ mobility features. Risk shifting, within the value chain of the alternative material or exposure pathways, must be explored and the outcomes kept transparent. A more comprehensive but also more resource intensive consideration of life cycle impacts is done with LCA discussed in more detail in 3. In LCA, impacts are assessed via quantitative methodologies and are implemented following the guidance of the ISO 14040 [46] and 14044 [47] norms. In AoA, the investigation of key attributes is, however, frequently only implemented as constituent of hazard, exposure, technical feasibility as well as the material’s ecosystem, rather than as an independent assessment element [41].

**Table 2:** List of existing resources supporting hazard and exposure assessments. NOTE: Some entries in this table are identical to the entries in other tables, due to the nature of the respective listings.

**Resource**	**Provider**	**Hazard, Exposure**	**Description**	**Link**
ChemForward	ChemForward	H	Method providing a cloud-based repository of chemical hazard assessment to support evaluations of alternatives	https://www.chemforward.org/
CompTox Chemicals Dashboard	United States Environmental Protection Agency (EPA)	H,E	Database for chemistry, toxicity, and exposure information for over a million chemicals. It includes chemical properties, environmental fate and transport, hazard, *in vitro* to in vivo extrapolation, exposure, bioactivity	https://comptox.epa.gov/dashboard/
C&L Inventory	European Chemicals Agency (ECHA)	H	Classification and labelling inventory database containing classification and labelling information on notified and registered substances received from manufacturers and importers	https://echa.europa.eu/information-on-chemicals/cl-inventory-database
eChemPortal	OECD	H,E	Physicochemical data source providing chemical hazard, risk, exposure and use information	https://www.echemportal.org/echemportal/
EPI Suite™-Estimation Program Interface (EpiSuite)	US Environmental Protection Agency	H,E	Suite of physical/chemical property and environmental fate estimation programs developed by EPA’s and Syracuse Research Corp. (SRC)	https://www.epa.gov/tsca-screening-tools/epi-suitetm-estimation-program-interface
European System for the Evaluation of Chemicals (EUSES)	European Chemicals Agency (ECHA)	H,E	Intrinsic exposure assessment data model that helps to carry out assessments of the risks posed by chemicals	https://echa.europa.eu/support/dossier-submission-tools/euses
GHS Column Model	Institute for Occupational Safety and Health of the German Social Accident Insurance (IFA)	H	Method that allows a quick comparison of substances and mixtures based on their hazard profile based on information found in the Material Safety Data Sheet or on the package labelling	https://www.dguv.de/ifa/praxishilfen/hazardous-substances/ghs-spaltenmodell-zur-substitutionspruefung/index.jsp
GreenScreen List Translator™	Clean Production Action	H	Hazard assessment screening tool, that provides a “list of lists” approach to quickly identify chemicals of high concern	https://www.greenscreenchemicals.org/assess/list-translator
GreenScreen Specified lists	Clean Production Action	H	Authoritative Lists provided by the GreenScreen List Translator	https://www.greenscreenchemicals.org/images/ee_images/uploads/resources/GreeScreen1.4-Annex11-1.18.pdf
GreenScreen Method	Clean Production Action	H	In addition to an evaluation of a chemical’s intrinsic human health and environmental hazards, the method provides a standardized score to facilitate communication throughout supply chains and within organizations	https://www.greenscreenchemicals.org/assess/method
Harmonized Classification and Labelling	European Chemicals Agency (ECHA)	H	Authoritative list containing all updates to the harmonised classification and labelling of hazardous substances	https://echa.europa.eu/information-on-chemicals/annex-vi-to-clp
OECD Substitution and Alternatives Assessment Toolbox (SAAT)	OECD	H,E	Compiles resources (tools, data sources case studies assessed by manufacturers, academic institutions, NGOs and government bodies) supporting consideration of hazard and exposures in AoA	https://www.oecd.org/en/topics/risk-management-risk-reduction-and-sustainable-chemistry.html
Pharos	NGO Healthy Building Network (Habitable)	H,E	Hazard and Exposure screening tool providing hazard, use, and exposure information on more than 200,000 chemicals used in the materials economy	https://pharos.habitablefuture.org/
PRIO	Swedish Chemicals Agency (KEMI)	H	Screening tool for identifying and prioritisation hazardous substances for substitution. It is a hazard-based model that is based on criteria. which refer to the inherent hazard properties of individual substances	https://www.kemi.se/prioguiden/english/start
PubChem	National Library of Medicine	H	Open chemistry database with chemical information from authoritative sources containing information about physical properties, biological activities, patents, health, safety, toxicity data	https://pubchem.ncbi.nlm.nih.gov/
PubMed	National Library of Medicine	H	PubMed supports the search and retrieval of biomedical and life sciences literature. Updated TOXLINE (now integrated in PubMed) content is available in PubMed or by searching PubMed using the search string: tox [sb]	https://pubmed.ncbi.nlm.nih.gov/
P2OASys	U.S.-based Massachusetts Toxics Use Reduction Institute (TURI)	H	Method to to compare the environmental, health and safety attributes of chemicals, formulated products and production process changes	https://p2oasys.turi.org/
Safer Consumer Products	Department of Toxic Substances Control (DTSC)	H	Hazard assessment screening list used by the California Department of Toxic Substances and Control’s Safer Consumer Products program	https://dtsc.ca.gov/scp/authoritative-lists/
SIN LIST	International Chemical Secretariat (chemsec)	H	Non-authoritative list of hazardous chemicals that are used in a wide variety of articles, products and manufacturing processes around the globe	https://sinlist.chemsec.org/
TEDX List	Endocrine Disruption Exchange (TEDX)	H	Non-authoritative list for endocrine disruptors (last update: 2018)	https://endocrinedisruption.org/interactive-tools/tedx-list-of-potential-endocrine-disruptors/search-the-tedx-list
Targeted Risk Assessment (TRA)	European Centre for Ecotoxicology and Toxicology of Chemicals (ECETOC)	E	Intrinsic exposure assessment data model that calculates the risk of exposure from chemicals for workers, consumers and the environment.	https://www.ecetoc.org/tools/tra-main/

#### 3.2.4 Assess performance and economic feasibility

Technical performance issues and the fitness-for-purpose boundaries in a product’s complex ecosystem are frequently the first surfacing obstacles to potential chemical substitutions. However, preset commensurate guidelines, technical and economic criteria, including associated decision rules and adopted supplementary regulatory policies from the assessment’s scoping phase can avert unwarranted early elimination and foster an unbiased navigation within set barriers. Different methodologies are implemented: The functional substitution approach prioritizes the question whether identified alternatives could fulfill the function without being critical to safety [48], [49], [50]. In contrast, less functional centred concepts favour strategies that focus on the application’s needs, implement less tightened technical and economic feasibility criteria with performance ranges instead of cut-off requirements and are content with sufficient performance [51], [52]. Performance assessment has to cover the dimensions of pure technical feasibility, such as in-place or accessible technology, as well as issues associated with legal, labour and supply chain criteria [41]. Economic feasibility assessment captures categories which can be subject to methods of cost evaluation, including economies of scale and market share, product innovation, cross-selling opportunities, new customer and revenue sources. Considering and forecasting the temporal development of associated costs and potentially monetizable opportunities within a reasonable payback period adds further complexity and needs the adoption of further expertise. Combining life cycle thinking in the stage of feasibility assessment could further inform the decision making and help to manage trade-offs, however the limited availability of data and methods to measure the socioeconomic footprint at all stages of the products value chain remains a challenge.

#### 3.2.5 Compare alternatives and make decisions

**Fig. 5:**
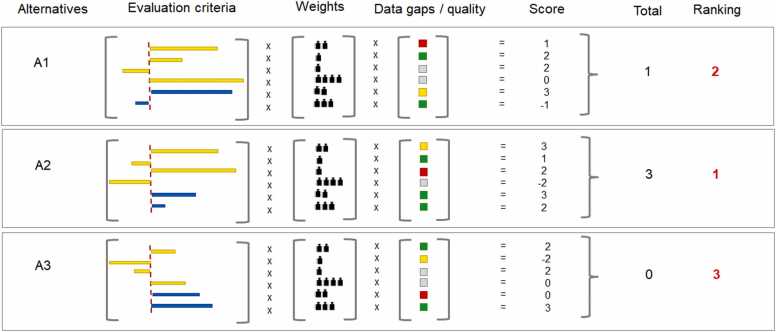
Schematic illustration of a basic implementation of a multiple-criteria decision analysis (MCDA): the evaluation criteria of the alternatives are assessed, relative to the benchmark (red dashed line): weights are determined by their relative importance via subjective judgment; data gaps / quality are considered by a rule-based classification within pre-set boundaries; the derived score for each criterion is integrated to an alternative’s overall total grading and final ranking.

Decision elements occur in all steps of the AoA, ring-fencing the options for a change, elimination or substitution of a SoC or its need in a specific application. Going beyond its role in risk assessment, decision making in AoA is an integrated path to support informed substitution, reduce adverse environmental footprint and optimize trade-offs [53]. All information collected about the dimensions (i) physicochemical properties, (ii) hazard characteristics, (iii) intrinsic exposure potential, (iv) technical and economic feasibility, (v) life cycle trade-offs has to be conflated into a multi-criteria comparison and decision-making approach which should lead to identify the most acceptable substitute. Remaining uncertainty and conflicting trade-offs need consideration in a transparent manner. Methodologies for decision making can be grouped by four dimensions: the decision function (comparative, ranking, none), the decision approach (sequential, simultaneous, hybrid), the decision methods/tools/rules (narrative, structural, analytical) and the role of weighting (qualitative, quantitative, implicit, none) [41], [54]. However, to facilitate a holistic view on sustainable decision making, incorporating aspects of data quality is an operational imperative. Knowledge gaps, the level of data confidence and degree of accuracy reflect a multifaceted additional dimension. High uncertainty in combination with complex trade-offs call for additional methodologies to improve the strength of decision outcomes. Many implementations of AoA lack transparency in regard to how uncertainty is handled and affects the decision [55]. A matrix of trade-offs (ranging from dominant alternatives to multiple trade-offs) vs. the grade of uncertainty (ranging from low to high uncertainty) may help to classify assessment results and to identify an appropriate decision strategy [56]. The existence of dominant alternatives and a low grade of knowledge gaps can waive the utilization of complex decision frameworks. However, in the presence of multiple trade-offs (i) a rule-based ranking or (ii) strict ordering of endpoints and assessment criteria or (iii) weighted scoring for each criterion ending up in a total score for alternatives is indispensable (Fig. 5). A structured and analytical methodology, multiple-criteria decision analysis (MCDA), helps to combine the information about specific endpoints with subjective judgment regarding their relative importance [57]. MCDA requires specialized skills and training. In exchange, it allows investigating the dependency of outcomes to the subjectively assigned preferences, essentially contributing to transparency, and fostering consistency. Missing data add the need for further rule-based considerations. These may include analytical methods of penalization, forced drop out of alternatives, further quantitative uncertainty analysis or an analytic loop back to earlier phases of AoA.

#### 3.2.6 From concept to practice

In summary, the following challenges, limitations, and opportunities result from the AoA concept when applying it to real-life applications:

• **Faulty launch:** The initiating scoping phase establishes the assessment approach’s boundaries and options for action, thus determining the range of potential outcomes. The consideration of all stakeholders’ settings and companies’ goals and principles requires the attitude from the person in charge that AoA is a multidisciplinary approach, thus, it depends on broad inclusiveness from the beginning which is frequently missing.
• **The drop-in replacement trap:** At the first glance, drop-in replacements may be a quick fix for a chemical substitution. However, drop-in replacements staying within the same class of molecules take the risk of being afflicted by the molecule class’s inherent adverse properties or being safe but tainted by the public’s negative perception whereas a broader way of thinking, promoting redesigning, questioning the need for use and change on a systemic level (material, product, technology) unfolds opportunities for innovation and strengthened competitiveness.
• **Risk shifting:** Life cycle thinking is frequently neglected or centred around the product use phase and human exposure routes. The assessment of a SoC’s life cycle impact, including its performance in complex ecological systems by specific methodologies, such as LCA, is mostly hampered by scarce data availability and the need for consideration of complex endpoints, such as a chemical’s mobility in environmental systems, its potential for biodegradability and bioaccumulation in specific organisms and trophic levels. However, informed substitution following the precautionary principle would mandate for systematically assessing the potential for risk shifting within ecosystems and value chains.
• **Lacking standards:** There are well-agreed standards (at least for chemicals, but less for materials or known mixtures like in advanced materials) for the assessment dimensions “human hazard” and “human exposure”, detaining endpoints, essential assays and threshold values to identify safety signals about potential adverse effects on human health. The level of agreement on critical elements in other dimensions, such as environmental sustainability, fate or social and economic impact, is by far less compelling. The degree of determination further diminishes regarding decision rules to be applied and how trade-offs are graded. At this point, a completely individualized perception and judgment may become influential to an assessment’s outcome.
• **Absence of evidence does not mean evidence of absence:** AoA relies on public domain data as well as on a company’s proprietary data sets. To be confident with AoA’s outcome, detailing data uncertainty in all possible dimensions (completeness, reliability, accuracy) is essential. Being transparent regarding how these elements are subject to evaluation and weighting and how these factors affect the final decision avoids prohibitive black-box methodologies. Without rule-based penalization of data uncertainty, some alternatives may be favoured by a better safety score; however, no evidence of hazard is not evidence of safety.

Going beyond AoA, the SSbD framework additionally endeavours to incorporate responsible R&I already in the design phase of the product development cycle, nevertheless, operating on the same design principles and employing the same or similar sets of tools and methodologies.

### 3.3 Use maps providing a systemic product view

When planning to bring safe products on the market, a risk assessment over the complete life cycle of a chemical or material is key. This is in line with steps 2 and 3 of the SSbD framework. Step 2 assesses the occupational safety and health aspects throughout the chemical/material's life cycle before final application. It involves identifying all production and processing steps, the chemicals used such as raw materials and processing aids, those produced during processes and their hazards. Step 3 evaluates the human and environmental impacts of applying the chemical or material, respectively. Like step 2, the use conditions will determine exposure likelihood, potential exposure routes (oral, dermal, inhalation), and related toxicity impacts on human health including service-life exposure and the environment [11].

To that end, understanding and estimating the exposure to the chemical or material during its entire life cycle is of paramount importance. Any activity where there is a potential of human or environmental exposure to a ChAM is defined in the current European chemicals legislation (REACH) as “use”. Use means any processing, formulation, consumption, storage, keeping, treatment, filling into containers, transfer from one container to another, mixing, and production of an article or any other utilization. Exposure scenarios is the term introduced by REACH to describe the operational conditions and risk management measures that ensure that these uses are safe during the life cycle of the chemical/material. Exposure scenarios summarize key information from risk assessments. They provide recommendations on how a chemical may be used and how to control exposure to workers and the environment to ensure the safe use. However, gathering all the necessary information that are crucial for risk and/or exposure assessments from customers and downstream users can be challenging and the exposure scenarios built by REACH registrants, based on assumptions end up being unrealistic.

Between 2013 and 2018, in the context of the chemical safety communication, ECHA and several stakeholder organizations developed the Chemical Safety Report/Exposure Scenario (CSR/ES) Roadmap [58], a plan for improving the content and use of Exposure Scenarios. A wider network called Expert Network of Exposure Scenarios (ENES) was established to develop use maps for realistic and sector specific exposure scenarios communication. This concept was developed to enhance the quality of information regarding uses and conditions of use communicated throughout the supply chain, as well as to improve the efficiency of this communication process. Typically, use maps are created by downstream user sector organizations, which gather information on the uses and conditions of use of chemicals within their sector in a harmonized and structured manner. To facilitate this, the ECHA established a “use map package” under the CSR/ES Roadmap [58]. By utilizing templates from the use map package, industry sectors generate use maps to collect the necessary information for conducting exposure assessments, including use descriptions and conditions of use. Registrants then leverage these use maps, developed by downstream user sectors, to prepare their chemical safety assessments (CSAs) under REACH, ensuring that the information reflects relevant and realistic uses and conditions. The application of use maps extends beyond REACH registrants, as they are also commonly employed by researchers and developers of new substances. By utilizing sector-specific information on use conditions, these developers can conduct comparative analyses of their substances against established market standards, including products or substances that have been available for several years. Currently there are map libraries available and updated accordingly, which include use descriptions and input parameters for assessing worker exposure (SWEDs), consumer exposure (SCEDs), and environmental exposure (SPERCs), provided by sector organizations for their typical applications. For instance, the Association of the European Adhesive and Sealant Industry (FEICA) has recently revised its use maps for adhesives and sealants across specific sector substance types [58]. These use maps offer a detailed overview of common applications within the sector, categorized by life cycle stage. Each application is identified by a specific use name and accompanied by pertinent market information. While the data provided in the use maps is tailored to a particular life cycle stage, it also serves as valuable information for realistically assessing the sustainability of newly developed substances or products.

## 4 S as in sustainability

In the field of sustainability assessment, LCA has become the de facto tool for conducting comparative and quantitative analysis of the environmental impacts of products and services. LCA is a standardized methodology that quantifies the impacts from birth to death (*i.e.* cradle-to-grave), with four distinctive stages: Goal & Scope Definition, Inventory Analysis, Impact Assessment, and Interpretation [46], [47]. In any ISO-complaint study, these four stages are followed in an iterative manner, having the Interpretation stage as the phase where results are revised, and re-iterations of the assessment are proposed, suggesting any LCA study is iterative by nature. For step 4 of the SSbD framework, the framework document stipulates that it “considers environmental impacts along the entire chemical/material life cycle by means of LCA, assessing several impact categories such as climate change and resource use” [11]. A few lines further down Caldeira *et al.* say that “LCA covers all the life cycle stages, as the determination of whether a chemical/material is SSbD or not, includes considerations of its functionality, *i.e.* intended use”. LCA is used within SSbD as it is the most well-established methodology for evaluating the (environmental) sustainability of novel materials and technologies according [59], [60]. Therefore - similar as for the first three steps we abstain here from further describing this methodology, but instead we elaborate how this quantitative tool could be applied from an early development stage in a so-called “forward-looking” or “prospective” manner. Readers interested in comprehensive details about the LCA methodology can consult Wolf *et al.* [61].

### 4.1 Taking a prospective perspective in LCA

With the growing number of LCA case studies related to emerging technologies such as in the field of nanotechnology, the term "prospective" (also sometimes called "*ex ante*") LCA gained considerably greater traction after initially appearing at the end of the 1990s [62]; as reported, for instance, in recent review studies from the Chalmers University of Technology in Sweden and the Fraunhofer Institute in Germany [63], [64]. As a further term, the term “anticipatory” LCA was introduced by Wender in the frame of his PhD, dealing with emerging photovoltaic technologies [65], [66]. Prospective and anticipatory LCA, both imply a temporal positionality of LCA, aiming at investigating how a current, (often immature) technology will evolve in a future time step while anticipating an increasing readiness level of the technology (*i.e.* including an increase of the technology maturity). Thereby, the term “Prospective LCA” always involves an increase in technology maturity in the future, which is not necessarily the case when speaking about “Anticipatory LCA”. “An LCA is prospective when the (emerging) technology studied is in an early phase of development (*e.g.* small-scale production), but the technology is modelled at a future, more-developed phase (*e.g.* large-scale production)” is one of the most comprehensive definitions for “Prospective LCA” [63]. On the other hand, “Anticipatory LCA” has been defined as a “forward-looking, non-predictive [LCA] tool that decreases the model uncertainty through inclusion of prospective modelling tools and multiple social perspectives” [65]. Arvidsson *et al.* [67] summarized in a recent review paper these various terms and definitions and concluded - in form of a recommendation to the LCA community – that “using the term prospective LCA, defined as ‘LCA that models the product system at a future point in time relative to the time at which the study is conducted’.” In addition, the authors of this review stress that due to the high relevance of the actual maturity of a technology, these aspects need to be clearly defined in any prospective LCA study. Core within PINK is the data issue on the level of the foreground system - *i.e.* the actual data of the investigated material. For related background data, *e.g.* the paper by Sacchi *et al.* [68] is showing how, based on integrated assessment models, suitable LCA databases for a future situation could be estimated with the help of the dedicated premise Python package. For more comprehensive information, readers can consult the paper by Langkau *et al.* [69], which provides structured and practical guidance to construct scenarios for prospective LCA.

### 4.2 Integrating LCA into SSbD at an early stage

**Fig. 6:**
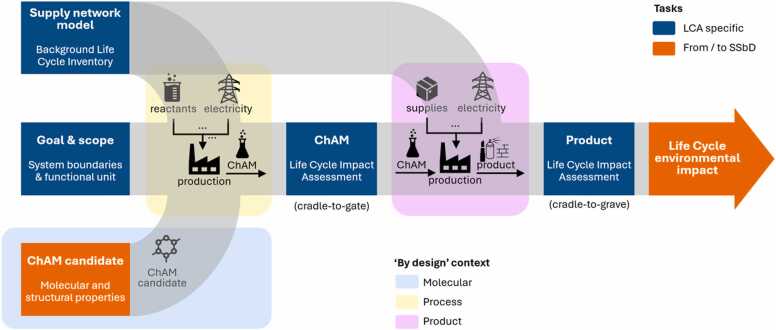
Methodological workflow of the step 4 of the SSbD framework. The flow shows the required tasks to conduct an environmental assessment of an innovative chemical and advanced material (ChAM) using LCA as the core methodological workflow. Blue boxes indicate required tasks proper to the standardized LCA methodology, while orange boxes depict tasks that come from or go to other steps of the SSbD framework. The light blue, yellow, and purple and light blue areas depict the “by-design” context in which the innovation can occur (light blue for molecular layer like by drop-in replacement in Fig. 2, yellow for process, and purple for product layer). A middle stage (cradle-to-gate) indicates the partial end of assessment when the ChAM application is not determined, while (cradle-to-grave) shows the end of a complete LCA when the ChAM application is known.

Combining sustainability and safety has been an issue already before the publication of the SSbD framework by the EC. Within the Horizon 2020 project NanoReg2, a model combining safe-by-design and sustainability was put into practice by applying LCA, human and environmental risk assessment, and an evaluation of the economic feasibility in a defined order [70]. Outcome of these activities was a decision tree – reported in [70] – that combines all three components in a nested, iterative process to produce competitive, safe, and sustainable materials, goods, or services. By enhancing the quality of the data gathered at each stage, this method, which is integrated into the stage-gate-model for SSbD, enables to lower the uncertainty associated with the assessment of risks and impacts. Within the SSbD guiding documents from the EC, the authors make a strong link between step 4 of the SSbD framework (*i.e.* the LCA study) and EC’s Product Environmental Footprint (PEF) methodology stipulating simply that “step 4 follows the PEF methodology” [11], [18]. In this sense, it is reasonable to consider that any practical implementation of the step 4 that follows this guideline will have a standardized LCA workflow as core and it will then incorporate SSbD considerations into it. One example of such methodological workflow is depicted in Fig. 6.

**Fig. 7:**
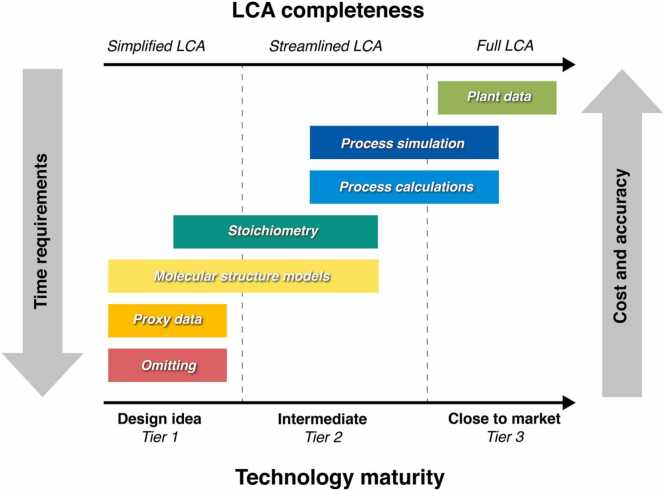
Relationship between the technology maturity (*i.e.* lower horizontal axis), cost and certainty of data generation methodologies (*i.e.* vertical axis), and the tiered LCA approaches as suggested in SSbD guidelines (*i.e.* upper horizontal axis). The location of the coloured blocks indicate which data generation method is suitable for each technology maturity and LCA completeness level.

Adapted from [71].

It is important to mention that the authors of the SSbD guiding documents stress that the LCA study needs to consider the actual state of knowledge of the innovation technology as well as the temporal aspect (the timeline). All of this affects all four steps that are distinguished within an ISO-compliant LCA study - leading to what Abbate *et al.* claim to be a “tiered approach of the increasing completeness of the (prospective) LCA” - going from a simplified LCA to full LCA [18]. In the frame of the development of innovative ChAMs, such a tiered approach can be presented in a slightly different manner. Namely, the completeness of an LCA depends on how much we know about the ChAM’s functionality or final usage, which, at the same time, is related with the availability and uncertainty of the data. This dependency and its relationship with the LCA tiered approach are depicted in Fig. 7. As it can be seen, when analysing products close to market, practitioners can rely on accurate data generation methods such as using plant or laboratory data, decreasing the uncertainty of the study and allowing the conduction of a “full LCA” (*i.e.* tier 3). On the contrary, when analysing products or ChAMs in the design phase, data might have to be generated using different techniques (*e.g.* proxy data or molecular structure models), increasing the uncertainty and limiting the study to be a “simplified LCA” (*i.e.* tier 1).

In Fig. 7, the different data generation methods [71] are scattered along the horizontal axis since they do not always correspond to a particular technology maturity or LCA level. In fact, while some methods are suitable to one particular tier, namely proxy data for simplified LCA or plant data for full LCA, others can be used for different LCA completeness levels (*e.g.* molecular structure models and stoichiometry for tiers 1 and 2, and process simulation and calculations for tiers 2 and 3). This variety of data generation techniques suggest that LCA practitioners will have to navigate in this spectrum rather than selecting from predefined approaches when conducting the step 4 in any SSbD implementation. Moreover, since SSbD is expected to be more useful when the ChAM is closer to the design idea rather than to the market, practitioners will have to deal with higher uncertainty and unknowns if higher benefits are aimed. In this sense, we can argue that LCA implementations in SSbD need to adopt a forward-looking perspective regardless of their approach nuances (*e.g.* prospective, anticipatory or similar) in order to address the challenges proper to early stages of innovation.

### 4.3 Machine learning and artificial intelligence - a key in early stages of applying forward looking LCA within SSbD

Any forward-looking approach has an intrinsic predictive component, which explains why, AI and Machine Learning (ML) are gaining more and more momentum [72]. The initial integration phase of ML in the field of LCA started around the beginning of the millennia, and it is now in a phase of complete integration (“intelligent sustainability phase”), with studies covering a plethora of different aspects, from inventory data gaps filling to toxicity characterization, or estimation of missing unit processes in life cycle inventories (LCIs) [73]. Several review studies have also dealt with this topic, highlighting the numerous promises, but also the potential limitations of AI applications in the sustainability field in general. These review papers analyse either the LCA field, regardless of the sector of application, a specific domain of application (*e.g.* buildings), or an entire industrial sector (*e.g.* the chemical sector) [74], [75], [76], [77]. A comprehensive example of such analysis can be the one provided by Liao *et al.* [77], in which they identified three main modes of adoption of AI in the chemical sector that to some extent could be generalized to other technology sectors.

The first one, termed AI-based, is the mode in which the quantitative relationships between the technical parameters (TPs) of the system at stake and the chosen performance indicators (PIs) that one wants to improve, are unknown. This implies that improving performance indicators mostly relies on trial and error or empirical knowledge. The TPs are controllable parameters (*e.g.* temperature and material flow rates for unit operations). PIs are performance indicators that depend on technical parameters (*e.g.* profits, energy consumption, and environmental emissions). These PIs are classified into five main aspects identified by the authors - *i.e.* economic aspects (*e.g.* net present value), energy-related aspects (*e.g.* energy efficiency), environmental aspects (*e.g.* total greenhouse gas emissions), safety and human factors (*e.g.* fault detection rate), as well as time-related aspects (*e.g.* reduced design, experiment, and computation time). With AI, PIs can be directly predicted or estimated based on existing datasets. Some examples reported by Liao *et al.* [77] include the AI-based predictive models for improving the process monitoring of unit operations [78], [79], [80].

In a second application mode, termed AI-optimized, the relationships between the TPs and PIs are known for the traditional system, but the system has not been fully optimized and AI-based optimization is used to improve the PIs. In this mode, the integration of ML models, particularly surrogate models with multi-objective optimization in chemical processes, has shown significant potential in optimizing complex systems. For example, Yang *et al.* [81] employed ML to optimize the CO_2_-to-olefins process, aiming to maximize light olefins yield and minimize carbon emissions. Similarly, Carvalho *et al.* [82] conducted sensitivity analysis and multi-objective optimization for the direct conversion of CO_2_ to DME, aiming to maximize DME yield and CO_2_ conversion.

The third application mode, termed AI-integrated, uses AI technologies to enhance the capabilities of traditional models for chemical production. In this mode, the relationship between TPs and PIs is also known as in the traditional model, and the integration of AI aims to extend the capability of the traditional model. For example, fuzzy logic has been used to enhance the fault tree analysis for the petrochemical industry by allowing for the estimation of failure probability with insufficient incident data [83], [84].

**Fig. 8:**
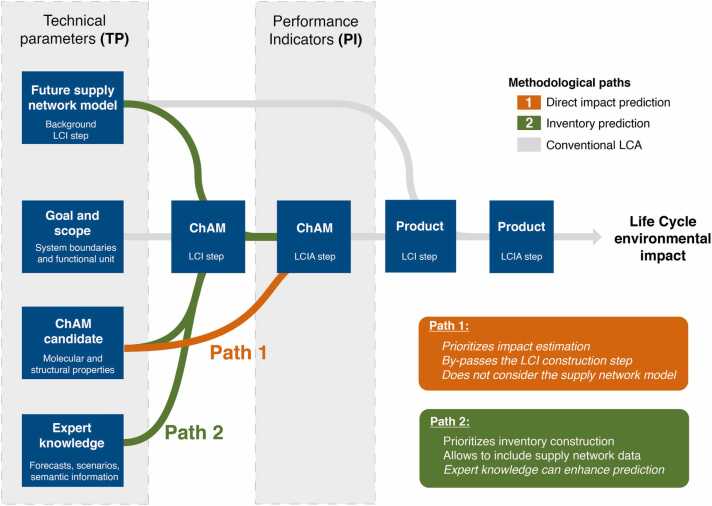
Illustration of the methodological workflow of step 4 of the SSbD framework for a low TRL innovative ChAM candidate: orange (*path 1*) and green (*path 2*) lines depict the two common methodological pathways as found in the literature. *Path 1* examples can be found in Zhu *et al.*[92], Song *et al.*[88], Sun *et al.*[89], Zhang *et al.*[90] and Kleinekorte *et al.*[91]. *Path 2* examples can be found in Hou *et al.*[86] and Zhao *et al.*[87].

These application modes are normally applied in a single instance in the existing studies, although some works have also used AI in more than one of these application modes [85]. The previously mentioned modes provide a general approach to discuss how AI can support the development of sustainable and efficient chemical processes at different levels. If this logic is extrapolated to a more LCA-specific context, supply chain configuration, ChAMs properties, and production data would take the place of the TP, and the LCA impacts would take the place of the PI. However, the literature shows that most of the AI applications in forward-looking LCA studies are AI-based (first mode) given that chemicals are analysed in an *in-silico* fashion, and production processes are yet unknown [86], [87], [88], [89], [90], [91]. From this point, it is easy to distinguish two possible methodological pathways for the SSbD context that have been explored in the AI-based LCA literature: impact prediction and inventory prediction. On the one hand, impact prediction (here termed path 1), maps a representation of the new ChAMs directly to aggregated life cycle impacts (*i.e.* orange line in Fig. 8). On the other hand, inventory prediction (path 2 hereafter), predicts intermediate steps (inputs in the LCI) to be later used in the calculation of the life cycle impacts (*i.e.* green line in Fig. 8).

#### 4.3.1 Path 1: advantages and limitations of an end-to-end approach

The main promise of path 1 is that it can allow the direct calculation of life cycle impacts of new chemical candidates, facilitating the screening of potential substitutes. Implementations of this path are data-driven, meaning that impact data of existing chemicals is required as a starting point. The common workflow consists of generating features by mapping a unique chemical identifier (*e.g.* Simplified Molecular Input Line Entry System (SMILE)) to numeric values represented by physical-chemical descriptors or an embedded space. These are then used as training data of conventional ML pipelines (*i.e.* data-processing, training, and validation) that map these features to the desired life cycle impact. Studies in the literature rely mostly on data from industrial partners and commercial life cycle databases, like Ecoinvent [93]. Moreover, literature shows a variety of training algorithms and approaches that can be aggregated into decision trees [89], Bayesian methods [91], ensemble [89], and neural networks [88], [89], [90], [92], [94], the latter being the most recurrent one.

An exhaustive list of examples of ML implementations in LCA can be found in a review made by Romeiko *et al.* [75], however some novel approaches, not included in that review, deserve to be highlighted. For instance, the first molecular structure-based predictive LCA tool based on artificial neural networks and basic molecular descriptors to estimate the environmental impact of chemicals was the FineChem tool [94], which was later enhanced in its FineChem2 version [90] by incorporating molecular graph information in a encoder-decoder architecture. Another interesting example is the one published by Kleinekorte *et al.* [91], where a more sophisticated application of ML algorithms is found. Here, the ML workflow also embedded expert-based information (*i.e.* flowsheet-based process descriptors) in addition to molecular descriptors to enable ML models to distinguish the impacts of chemicals produced by different processes. They presented a predictive LCA framework made up of an encoder-decoder neural network and a Gaussian Process Regression (GPR), which enabled process-specific prediction of the global warming impact scores of organic molecules.

**Fig. 9:**
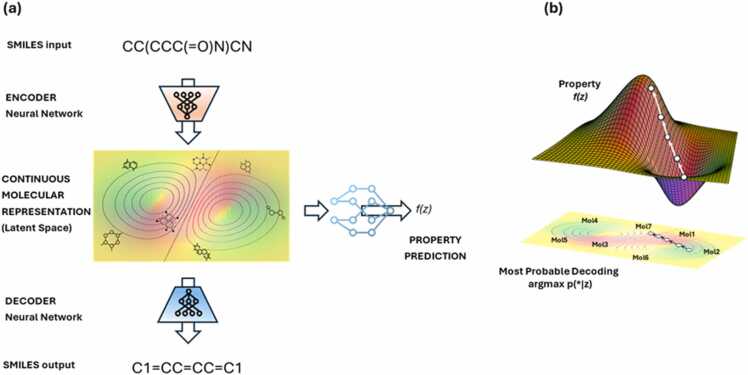
Composite figure providing (a) a diagram of the autoencoder used for molecular design, including the joint property prediction model and (b) an illustration of the gradient-based optimization in continuous latent space.

Adapted from [95].

While the current literature is leveraging the power of encoder-decoder architectures to generate embedded spaces, there is still room for exploring how embedded spaces could be used for tasks beyond the prediction of impacts. For instance, starting from a discrete molecular representation, such as a SMILE string, an encoder model can convert each molecule into a continuous molecular representation in a latent space. Then, a surrogate model f(z) can be trained to predict a property of interest (*e.g.* global warming impact) based on their latent representation z. f(z) can later be optimized with respect to z to find new latent representations expected to have better values of the desired property. These new latent representations can then be decoded into SMILES strings, from which their properties can be tested empirically. The concept, which can be generalized to many applications related to finding chemical substitutes for substances of concern, is illustrated in Fig. 9 [95]. While not necessarily novel in the design of chemicals, this generative approach can represent an opportunity for sustainability research since it could allow gradient-based optimization, interpolation, or simply exploration of novel chemicals considering their life cycle impacts as desired property.

Despite the successful application in approximating life cycle impacts of some chemicals, path 1 models tend to become black boxes with low interpretability. Moreover, they can have narrow applicability domain because of limited training data, which restricts their application to basic chemicals with simple molecular structures. In this sense, more LCA data considering more ChAMs (*e.g.* nanomaterials) is required, and algorithms should wean off identifiers like SMILEs towards approaches capable of parsing complex molecular structures (*e.g.* polymers).

Finally, one of the most important limitations of path 1 models is that the aggregated life cycle impacts embed the supply chain information and corresponding database assumptions, which responds to specific temporal and geographical contexts. For instance, most of the studies use different versions of Ecoinvent to calculate environmental impacts which may contain updated information in terms of markets or electricity mixes. This implies that the different models in the literature predict different environmental impacts for the same chemical. Additionally, given this end-to-end nature, practitioners are not capable of introducing additional information to explore different prediction scenarios, exposing the need of more flexible approaches, such as the one proposed in path 2.

#### 4.3.2 Path 2: The potentials of a flexible approach

Path 2 models in the literature are not abundant, and they are far from being a mainstream forward-looking LCA approach. The promise of path 2 is that it can allow the prediction of the intermediate steps of the LCA, namely the LCI, which more easily allows incorporating new information when it becomes available and can then be used as baseline in multi-scenario analysis (*e.g.* process optimization). In the LCA literature, only two studies have focused on exploring this path by treating the life cycle data as a graph of interconnected processes and predicting the missing links and values when only little information is provided [86], [87]. While not specific for chemicals, these two studies included chemicals as part of the training dataset. Zhao *et al.* [87], which is an enhancement of Hou *et al.* [86], proposed a two-steps model consisting of an initial prediction of potential links (*i.e.* binary classification task), followed by the prediction of the flows of the predicted links (*i.e.* regression task). Taking a chemical as an example, the resulting prediction then consisted of the set of processes (*i.e.* inputs) and flows (*i.e.* values) required to produce one unit of the desired chemical. This data could then be plugged into any LCI model to proceed with the impact calculation as it were part of any conventional LCA study.

While highly promising, this path has not been explored more in the literature. We argue that the absence of attempts could be explained by the difficulty of predicting links and values when no data, but the chemical candidate is available. In fact, Zhao *et al.* [87] results indicated that reasonable predictions can be obtained when no less than 10 non-zero values are known. Additionally, this approach is only valid when dealing with structured data formats (*e.g.* graph data), such as the ones provided by Ecoinvent, meaning that any unstructured data entry, such as expert knowledge, diagrams, equations, or semantic information would need to be converted first into LCI data in a valid Ecoinvent-compatible format. We argue that the integration of such unstructured data into the modelling exercise, both in the form of process-based information (flowsheet-based models) and expert knowledge contained in semantic or metadata information, can represent a breakpoint in forward-looking LCA modelling. The premise is that this sort of information can be retrieved from several sources, such as the explicit process description of Ecoinvent processes, lab logbooks, reports, etc, considerably increasing the knowledge base and allowing the human intervention in the impact calculation. This integration, however, may be constrained by the limited capacities of ML algorithms to deal with multiple data structures simultaneously.

Current developments in AI, namely large language models (LLMs), may shed light on the possible directions of path 2. More specifically, unstructured information could be parsed using transformer-based models (*e.g.* BERT) to provide embeddings of the data to then be plugged into artificial neural networks [96], [97], [98]. To limit the drawbacks related to low-representativity (for the specific problem at hand) of the data used during the pretraining step of the LLMs, the learning task can be enhanced by applying retrieval augmented generation (RAG) techniques, that can introduce new information during inference that was not present during the pre-training process [99]. While not in the same way as described above, the literature has shown attempts to exploit the power of LLMs for LCA purposes. For instance, EcoScan is a commercial tool [100] capable of automating the construction of LCI data of bills of materials that are built from semantic descriptions through the use of chatbots. In another example, Balaji *et al.* used LLMs in combination with RAG to match the user’s prompt to the most similar Ecoinvent process.

In the context of SSbD, leveraging semantic information via LLMs could address the path 1’s inability to include different synthesis routes and production processes since they cannot be reflected in the molecular structures, and it is therefore not captured by the model. In this sense, one important modelling direction to take in the future is therefore the incorporation in the models of the information concerning the different synthesis routes and production processes to achieve more accurate predictions. The use of semantic information can facilitate this process, because process descriptions in textual form can help in identifying the exact synthesis route taken, or at least the best proxy for it [97].

## 5 By design

As it was previously presented, developing ChAMs considering available information for all dimensions (*i.e.* functionality, safety, and economic, environmental, and social sustainability) at every stage of process is a complex task. In fact, open issues like data integration, balancing of design goals and (AI-based) decision support still require further addressing if we want to create highly functional, safer and more sustainable ChAMs truly BY-DESIGN. As highlighted in the 2, the holistic view on the complete material life cycle and the full, partly interlinked, and circular value chains is inseparable from SSbD. The extended frameworks described above support this view but also lead to technical challenges. For example, use maps integrate the product and use phase into safety assessment. LCA, as its name implies, covers the full life cycle but needs to be further developed to support decision making at early material development stages based on very limited data. Due to the largely independently developed regulatory framework for safety assessment, industry standards for environmental and social sustainability assessment as well as material characterisation and modelling for functionality optimization, coverage of the life cycle stages and how they are considered in the final evaluation are implemented differently in the dimensions and further harmonization of data and new approaches to reuse and balance information across dimensions are required for making SSbD manageable and less time consuming.

Considerable efforts are being devoted at European level towards the development of tools to support the implementation of SSbD, with 45 projects with focus on SSbD funded by the European Commission at the time of writing this perspective article [13] with more to come. The digital and computational dimension, essential for pushing safety and sustainability into the earliest stages of material development, is prominent in several of these funded projects including the PINK project. PINK proposes the development of a generally applicable, automated approach integrating various modelling methodologies to provide the input for comprehensive and effective AI-driven decision support following a multi-objective optimization approach balancing functionality, costs, safety, and sustainability. Full details on the resulting digital PINK open innovation platform and the underlying semantic interoperability framework for data and modelling are available from the recently published PINK Innovation Report [101]. The principles that have inspired the whole idea-generation of PINK stem from the frameworks, methodologies, and views from the industry standpoint described in the previous sections to enable an actionable and operational platform that allows a practical implementation of SSbD of new ChAMs.

It cannot be stressed enough that effective, efficient and even more important, trustworthy data and knowledge provision, management and integration is key. However, we also stress here again that all required data to perform a complete SSbD assessment cannot be generated for all possible, potentially only virtually existing candidates considered during early-stage development and are also not needed to make decisions at these stages. For this reason, PINK is following a tiered approach, where each development cycle starts with mining and creating data on the functionality, safety, and sustainability, as well as cost/economic feasibility, which will be provided simultaneously as input for the decision support workflow. Data will be generated using modelling and simulation techniques with high throughput but compromising achievable confident levels in the early development stages, which will then be replaced with more reliable computational and experimental data when moving to later stages, in which the number of candidates was already considerably reduced. For example, in the cases where - on lab-scale or small pilot-scale level - first data concerning the synthesis process of a substance are becoming available, such data can be used as a starting point for the sustainability assessment. This data is combined with adequate computational upscaling procedures to calculate a first estimation of the environmental impacts of a (future) full-scale production, for instance in line with [102].

Knowledge graphs are being used in PINK and serve as a unified representation of information, enabling the assessment of environmental impact and safety of chemical substances to identify new patterns and relationships that are not evident using traditional analysis methods. The first type of knowledge graph is implemented in the PINK Knowledge Base, which will index all relevant data and modelling resources, which can then be integrated into the automated workflows and the decision process based on the selection made by the user. The second type of knowledge graphs contains nodes that represent ChAMs, as well as other types of nodes that provide additional context about physical, biological, and environmental properties, effects on biological systems following the AOP framework, as well as societal and economic indicators usable for reasoning during the decision-making process and for documenting this process.

Yet another challenge remains for moving from relative (safer, more sustainable) to absolute criteria (safe and sustainable) in SSbD and, thus, also in PINK, which is related to the investigation of the link between life cycle environmental impacts and related market external biophysical targets, like planetary boundaries. Although this is one of the points recommended by a recent overview of the research on SSbD at European level [13], unfortunately none of the currently EU-funded projects on SSbD addresses aspects related to setting absolute environmental sustainability targets and benchmarking, more specifically for chemical pollution (novel entities). This is certainly due to the intrinsic complexity of planetary boundaries quantification and downscaling to product level, but probably also to the fact that absolute environmental sustainability assessments could be better suited for large-scale systems than smaller-scale chemical productions [103]. Nonetheless, if at least a set of indicators to bridge the gap between early-stage development phases of a product and expected final positioning with respect to the planet's carrying capacity will be developed, this could help industry moving towards absolute sustainability, until a full absolute sustainability assessment framework will be available.

In summary of this first aspect of by-design, central to the PINK R&I approach of creating software- and model-assisted integrated workflows for the provision of SSbD-based ChAMs are in fact: (i) the harmonization of data including the corresponding terminology, and (ii) the standardization of the FAIRification approaches as part of the interoperability framework. This ensures the consolidation of hitherto siloed data (*i.e.* siloed into the separate use categories: (a) materials, (b) safety, and (c) sustainability modelling). The resulting ChAMs data and tools infrastructure bridges the communities of safety, sustainability and materials modelling, allows integration of modelling in an efficient and effective way into the PINK decision support and could be considered as a prototype for a European materials data ecosystem.

For this decision support as the second aspect of by-design, PINK uses generative AI to explore the vast chemical space and discover the most suitable ChAMs for further development. By incorporating development-stage-specific property constraints, the decision support workflow will generate novel structural fingerprints, evaluate their chemical feasibility, and propose new promising ChAMs designs for the specific application. The models will be constructed using *in silico* characterization of molecular structures and complemented by experimental features as necessary for the predictive models' performance. This way, PINK follows the recommendations for the methodological developments for SSbD that were suggested already in 2019 by a seminal Non-Paper published by a working group of public and private sector experts [104].

The workflow proposed for PINK’s decision support system was already described in the PINK Innovation Report [101] and will only be summarised here shortly:

1. Define the criteria for the four categories (functionality, cost-efficiency, safety, and sustainability) that will be used to optimize and evaluate the design candidates according to the tier level corresponding to the actual material development stage.
2. Analysing the information space by search the knowledge graph for similar ChAMs to the design candidates using AI techniques such as graph or fingerprint similarities.
3. Depending on the volume of available data, use read-across methods or machine learning QSAR and QSPR models to predict functionalities and SSbD properties of similar ChAMs.
4. Use mechanistic multi-scale simulations to predict functionalities and interactions between selected candidates and their environment that cannot be predicted using data-driven models.
5. Conduct LCA for selected candidates to evaluate their environmental impact over their entire life cycle, including production optimization, use, and end-of-life.
6. Perform real-time evaluation and comparison of candidates based on their predicted functionalities, LCA analysis and SSbD characterisation. Utilising knowledge-graph-based reasoning and visualisations tools to guide the identification of the most important criteria to be used in the selection of candidates to proceed with.
7. Leverage the power of generative and causal AI combined with multi-objective optimization approaches to explore new and innovative ChAM design options, beyond the limitations of existing data. By incorporating property constraints, the decision support workflow will generate novel structural fingerprints, evaluate their chemical feasibility, and propose new promising candidates for the specific application.
8. Present the final shortlist of selected candidates. A multi-decision support algorithm will rank the ChAMs included in the final list, according to the optimization criteria. Furthermore, at this stage the DSS will provide recommendations and tools for additional computational and experimental testing to increase confidence in the predictions and perform risk analysis on the most promising candidates.

The first implementation of multi-objective optimization approach in step 7 will apply optimization of molecular sequences (*e.g.* based on SMILES) or graph representations of ChAMs. Three solutions are possible here:

1. Forward solution where we generate similar substances and pass them through the matrix of models: These compounds can be explored for their potential to exhibit similar functional properties, while providing an opportunity to design safer and more sustainable alternatives. Various computational tools and methods are available to facilitate the generation of these similar compounds, relying on molecular similarity and the exploration of chemical space. One approach to generating similar compounds involves the use of molecular similarity tools, such as PubChem Similarity Search, which compares molecular fingerprints of compounds and identifies those with similar structural features, by utilizing similarity metrics, such as the Tanimoto coefficient. In addition to similarity-based approaches, isomer generators, like Maygen [105], Open Molecule Generator (OMG) [106], and Surge [107], can be employed to create novel molecules that resemble the substance of concern while adhering to predefined constraints. Once similar compounds are generated, they are passed through the matrix of models available in the PINK platform. These models assess the compounds based on SSbD indicators. The results are consolidated into reports that summarize the model predictions with respect to SSbD criteria, and the findings are presented in a visualized form. This visualization enables the designer to evaluate the suitability of the generated compounds and make informed decisions regarding the safer and more sustainable alternatives.
2. Stochastic evolutionary algorithms: These are optimization techniques that rely on random processes to find solutions to complex problems, where the solution space is large, non-linear, or poorly understood. Unlike deterministic algorithms, which follow a fixed sequence of steps, stochastic algorithms use randomness in their search strategy, allowing them to explore a broader range of solutions and avoid getting stuck in local optima. One popular type of stochastic algorithm is the Genetic Algorithm (GA), inspired by natural selection [108]. In GAs, potential solutions are represented as individuals in a population, and the algorithm evolves the population over generations using selection, crossover (recombination), and mutation. The fitness of each solution is evaluated based on an objective function, and the best solutions are selected to create new generations. Another well-known stochastic algorithm is Simulated Annealing (SA), which is inspired by the physical process of annealing in metallurgy, where a material is slowly cooled to reach a stable state. SA starts with an initial solution and explores the solution space by making random changes. If a new solution is better, it is accepted; if it is worse, it can still be accepted with a certain probability that decreases over time, mimicking the gradual cooling process. This probabilistic acceptance of worse solutions helps SA avoid getting trapped in local optima and improves the chances of finding the global optimum. Stochastic algorithms have excellent applications in molecule and material design, where multiple factors must be optimized simultaneously [109]. In a multi-objective algorithm, the optimization process handles multiple conflicting objectives, related to functionality, safety, and sustainability. One approach is to combine these objectives into a single composite objective function, typically by defining a weighted sum of individual objectives, where each objective is assigned, a weight based on its importance. The weights allow the user to control the trade-offs between the conflicting goals, guiding the evolution towards solutions that best meet the overall design criteria. However, determining the appropriate weights is crucial, as they can significantly influence the outcome. Alternatively, instead of focusing on a single objective function, a multi-objective algorithm can evaluate and balance several fitness functions that represent different design goals separately. Pareto optimality is a key concept in this case, where solutions are considered optimal if no other solution is better in all objectives. As the algorithm progresses, it generates a set of diverse, non-dominated solutions that form the Pareto front, providing a range of possible designs for the researcher to choose from [110]. A third option is a hierarchical setting, where the different objectives are ranked in an order of interest [111].
3. Generative AI: Generative AI Generative AI offers innovative strategies for designing novel chemical substances [112]. Architectures such as Generative Flow Networks (GFlowNets) [113], variational autoencoders (VAEs) [114], and generative adversarial networks (GANs) [115] are increasingly used to explore chemical space and optimize the generation of valid, synthesizable substances.

• VAEs learn latent representations of molecular structures, allowing them to generate new molecules with desired properties by sampling from this learned space. When combined with reinforcement learning or predictive models, VAEs can be directed to produce molecules that meet specific SSbD thresholds.
• GANs employ a dual neural network system, where a generator proposes new molecular structures and a discriminator evaluates them, iteratively improving the quality and relevance of generated compounds.
• GFlowNets are a type of neural networks designed to generate diverse and high-quality samples from complex distributions. GFlowNets aim to model the probability distribution of a set of outcomes by learning a flow that can generate sequences of discrete objects, like chemical structures.

In the PINK multi-objective optimization framework, we will explore how generative AI can be tailored to synthesize alternative substances to those of concern, while considering SSbD factors such as safety, sustainability, and performance. PINK is already applying such algorithms for the production process optimization. Such an optimization is a key component for SSbD on the product or even system level and can result in reduced emissions and energy consumption, protects workers from exposure, improves production efficiency, minimises product variability, and ensures that products meet design specifications. Advanced data-driven, multi-objective algorithms such as Bayesian optimization and the Thompson sampling for efficient multi-objective optimization (TSEMO) method [116] are used to determine the optimal conditions in the production processes that balance yield, product consistency, and environmental factors. This combination with real-time monitoring and data analysis through onboarding of third-party tools into the PINK platform will provide a robust workflow to control and optimize the production processes of the new substances, promoting sustainability by reducing waste, improving product quality and reliability, and minimising environmental impact.

The need for harnessing the potential of AI tools and approaches to foster, or even to allow the implementation of the SSbD framework, has been clearly highlighted in recent literature [117] and PINK is perfectly integrated in this line of research. PINK’s AI-based modelling pipeline will be leveraged to generate reliable estimates of relevant data to conduct LCA of the selected ChAMs to evaluate their environmental impact over their entire life cycle, including production optimization, use, and disposal.

While the methodologies described showcase robust capacities for assessing the environmental, safety, and socio-economic dimensions especially close to product launch, they also underline still existing inherent limitations, particularly in handling uncertainties at early innovation stages stemming from limited data availability and reliance on predictive modelling. These uncertainties emphasize the need for iterative refinement of the adaptive decision support system. To bridge these gaps, the validation of the proposed methodologies on specific examples will play a pivotal role, providing tangible insights and benchmarks to enhance the confidence and applicability of the SSbD framework in real-world scenarios. This iterative process of validation and adjustment will ensure the methodologies not only align with theoretical expectations but also demonstrate practical relevance and reliability.

## 6 Conclusions

Taking an industry-focused approach, we have laid out perspectives for R&I in the direction of integrating and balancing the relevant SSbD dimensions safety, sustainability, and performance prioritizing topics in relation to activities within the PINK project that endeavours to provide multi-objective optimization procedures for early innovation stages less in an iterative than in a more parallel manner. The SSbD Framework in its current version proposes 5 steps. Even if the word “step” implies a consecutive execution, the SSbD framework opens the possibility to run them in parallel. First learning from this testing as well as expertise from SSbD material development reported by industry shows that the current set of criteria needs to be further refined and SSbD shows to be most beneficial if the steps are executed in parallel and not in series. Crucial for computational approaches *per se* is the data issue. Thus, for early-stage cases of innovation, AI-based forward-looking procedures in LCA build the core of the sustainability section presented. In the cases where - on lab-scale or small pilot-scale level - first data concerning the synthesis process of a substance are becoming available, such data can be used as a starting point for more refined assessment, and multi-objective optimization approaches can start incorporating such methodologies.

**Abbreviations**

**Table:** 

R&I	Research and innovation
SSbD	Safe and Sustainable by Design
SoC	Substances of concern
AoA	Assessment of Alternatives
LCA	Life cycle assessment
EU	European Union
CSS	Chemicals Strategy for Sustainability
ChAMs	Chemicals and advanced materials
EC	European Commission
JRC	Joint Research Centre
PFAS	Per- and polyfluoroalkyl substances
SVHC	Substances of very high concern
CMR	Carcinogenic-mutagenic-reprotoxic
PBT	Persistent, bioaccumulative, toxic
vPvB	Very persistent, very bioaccumulative
PMT	Persistent, mobile, toxic
vPvM	Very persistent, very mobile
ED	Endocrine disruption
CEFIC	European Chemical Industry Council
PSA	Product Sustainability Assessment
WBCSD	World Business Council for Sustainable Development
TRL	Technology Readiness Level
DSS	Decision Support System
NAMs	New Approach Methodologies
IATA	Integrated Approaches to Testing and Assessment
AOPs	Adverse Outcome Pathways
GHS	Globally Harmonized System
DA	Defined Approaches
TG	Test Guidelines
SIRs	Standard information requirements
PoD	Point of Departure
UN	United Nations
SMEs	Small and Medium Enterprises
TURI	Massachusetts Toxics Use Reduction Institute
ECHA	European Chemicals Agency
SAAT	Substitution and Alternatives Assessment Toolbox
SUBSPORTplus	Substitution Support Portal
BAuA	Federal Institute for Occupational Safety and Health
EPA	Environmental Protection Agency
SCIL	EPA’s Safer Chemical Ingredients List
QSAR	Quantitative Structure Activity Relationship
OECD	Organization for Economic Co-operation and Development
EpiSuite	Estimation Program Interface
EUSES	European System for the Evaluation of Chemicals
IFA	Institute for Occupational Safety and Health
SAAT	OECD Substitution and Alternatives Assessment Toolbox
Habitable	NGO Healthy Building Network
KEMI	Swedish Chemicals Agency
DTSC	Department of Toxic Substances Control
Chemsec	International Chemical Secretariat
TEDX	Endocrine Disruption Exchange
TRA	Targeted Risk Assessment
ECETOC	European Centre for Ecotoxicology and Toxicology of Chemicals
MCDA	Multiple criteria decision analysis
REACH	European chemicals legislation
CSR	Chemical Safety Report
ES	Exposure Scenario
ENES	Expert Network of Exposure Scenarios
CSAs	Chemical safety assessments
SWEDs	Specific Worker Exposure Descriptions
SCEDs	Specific Consumer Exposure Determinants
SPERCs	Specific Environmental Release Categories
FEICA	Association of the European Adhesive and Sealant Industry
PEF	Product Environmental Footprint
ML	Machine Learning
LCIs	Life cycle inventories
TP	Technical parameters
PIs	Performance indications
SMILE	Simplified Molecular Input Line Entry System
GPR	Gaussian Process Regression
LLM	Large language models
RAG	Retrieval Augmented Generation
OMG	Open Molecule Generator
GA	Genetic Algorithm
SA	Simulated Annealing
GFlowNets	Generative Flow Networks
VAEs	Variational autoencoders
GANs	Generative adversarial networks
TSEMO	Thompson sampling for efficient multi-objective optimization

## CRediT authorship contribution statement

**Gallegos Gustavo Martin Larrea:** Writing – original draft, Visualization, Methodology, Investigation, Formal analysis. **Norbert Hofstätter:** Writing – review & editing, Writing – original draft, Visualization, Investigation. **Sabine Hofer:** Writing – review & editing, Writing – original draft, Visualization, Investigation. **Nico Watzek:** Writing – original draft, Visualization, Investigation. **Benjamin Punz:** Writing – original draft, Visualization, Methodology, Formal analysis. **Karin Wiench:** Methodology, Investigation, Formal analysis. **Wibke Lölsberg:** Visualization, Methodology, Formal analysis. **Antonino Marvuglia:** Writing – review & editing, Writing – original draft, Visualization, Supervision, Methodology, Investigation, Funding acquisition, Formal analysis, Conceptualization. **Roland Hischier:** Writing – review & editing, Writing – original draft, Visualization, Methodology, Investigation, Formal analysis. **Martin Himly:** Writing – review & editing, Writing – original draft, Visualization, Supervision, Methodology, Investigation, Funding acquisition, Conceptualization. **Irantzu Garmendia Aguirre:** Writing – original draft, Methodology, Formal analysis. **Wendel Wohlleben:** Investigation. **Haralambos Sarimveis:** Methodology, Investigation, Formal analysis. **Nikolakopoulos Athanassios:** Writing – original draft, Investigation. **Christian Seitz:** Writing – review & editing, Visualization. **Anna Costa:** Writing – original draft, Methodology, Formal analysis. **Thomas E. Exner:** Writing – review & editing, Supervision, Methodology, Investigation, Funding acquisition, Formal analysis. **Steffi Friedrichs:** Writing – review & editing, Visualization.

## Funding

PINK has received funding from the European Union’s Horizon Europe Research and Innovation programme under grant agreement No. 101137809, the Swiss State Secretariat for Education, Research and Innovation (10.13039/100004152SERI) under grant REF-1131-52302. Further, this work was supported by the SmartCERIALS project of the Austrian Research Promotion Agency (FFG, Grant No. 890610).

## Declaration of Generative AI and AI-assisted technologies in the writing process

No AI assistance was used for drafting this document.

## Declaration of Competing Interest

The authors declare to have no conflicting interests with the content of the study, have read and revised the manuscript carefully, agreed to its submission, and accepted their responsibility for the content.

## Data Availability

Not applicable.
